# Prevalence and influencing factors of liver injury in naïve patients with HIV/AIDS in Nanjing from 2005 to 2022: Cross-sectional study

**DOI:** 10.1097/MD.0000000000041261

**Published:** 2025-05-30

**Authors:** Nawei Yu, Xiaoyun Di, Zihao Xia, Jingli Peng, Mingli Zhong, Mengqing Li, Hongjing Guan, Chen Chen, Rentian Cai, Hongxia Wei

**Affiliations:** a Department of Infectious Disease, The Second Hospital of Nanjing, Affiliated to Nanjing University of Chinese Medicine, Nanjing, Jiangsu Province, China; b Department of Infectious Disease, The School of Public Health of Nanjing Medical University, The Second Hospital of Nanjing, Nanjing, Jiangsu Province, China.

**Keywords:** ART-naïve, HIV/AIDS, liver injury

## Abstract

To investigate the prevalence of liver injury and the factors influencing severe liver injury in antiretroviral therapy (ART)-naïve patients with human immunodeficiency virus (HIV) infection. ART-naïve HIV-1 infected patients who visited the outpatient department of the Infection Department of the Second Hospital of Nanjing between January 1, 2005, and May 31, 2022, were included in the study. The clinical data of patients with baseline liver injury were retrospectively collected. Liver injury was classified as grade 1, 2, 3, or 4 according to the severity scale of adverse events in adults and children with acquired immunodeficiency syndrome (AIDS), based on the levels of alanine aminotransferase (ALT), aspartate aminotransferase (AST), and total bilirubin (TBIL). A total of 982 patients were included in the analysis. The overall prevalence of liver injury (grades 1–4) was 19.3% (982/5099), with grade 1 liver injury (75.9%, 745/982) be the most common. Multivariate logistic regression analysis revealed that high-density lipoprotein cholesterol (HDL-C) was a protective factor against severe liver injury (odds ratio [OR] = 0.28, 95% CI = 0.08–0.99, *P* = .048). Conversely, hepatitis B virus (HBV) infection (OR = 4.02, 95% CI = 1.82–8.88, *P* = .001) elevated gamma-glutamyl transpeptidase (GGT, OR = 1.04, 95% CI = 1.01–1.06, *P* = .004), and elevated levels of lactic dehydrogenase (LDH, OR = 1.03, 95% CI = 1.01–1.04, *P* = .002) were identified as independent risk factors for severe liver injury in newly treated HIV/AIDS patients. Liver injury is prevalent among HIV/AIDS patients who have not initiated ART. HDL-C, HBV infection, GGT, and LDH are significant factors influencing the severity of liver injury.

## 1. Introduction

With the widespread use of antiretroviral therapy (ART), the life expectancy of human immunodeficiency virus/acquired immunodeficiency syndrome (HIV/AIDS) patients has significantly increased,^[1]^ and related mortality has decreased.^[2]^ However, the aging of HIV/AIDS patients, long-term immune activation, persistent inflammatory state, immune senescence and drug side effects have been reported.^[3]^ Consequently, non-AIDS-related mortality is on the rise.^[4,5]^ Among non-AIDS-related deaths, liver disease-related deaths (13%–18%) have become the most common cause.^[6]^ Even in HIV-infected patients who are well controlled by ART, liver-related death remains the leading cause of non-AIDS-related mortality.^[7]^ The age- and sex-standardized mortality from liver disease is 3.7 times higher in HIV/AIDS patients compared to the general population (95% CI = 3.3–4.2).^[8]^

Currently, there is a lack of uniform standards for defining liver injury both domestically and internationally.^[9,10]^ Consequently, the reported prevalence of liver injury varies significantly across different studies, depending on study design and population.^[9,11]^ In studies focusing on HIV monoinfection, the prevalence of liver injury ranges from 6.4% to 32%.^[12,13]^ This prevalence is higher among patients co-infected with hepatitis C virus (HCV) or hepatitis B virus (HBV), with reported rates ranging from 13% to 51.8%.^[14]^ Previous research has indicated that the prevalence of severe liver injury ranges from 2.2% to 15.0%,^[15,16]^ predominantly among treated patients, suggesting a potential influence of ART toxicity. However, severe liver injury has also been observed in some ART-naïve patients,^[17]^ although studies in this area are limited and the factors contributing to severe liver injury in these patients remain unclear.

Serum alanine aminotransferase (ALT) level is the most specific indicator for monitoring liver injury.^[12]^ Multiple studies have demonstrated that elevated ALT levels are associated with increased liver-related mortality.^[18,19]^ Elevated ALT is common among HIV/AIDS patients; however, approximately half of HIV/AIDS patients receiving antiretroviral therapy are asymptomatic,^[20]^ which often leads to insufficient attention from clinicians. ALT levels are frequently assessed in conjunction with other variables such as aspartate aminotransferase (AST), alkaline phosphatase (ALP), gamma-glutamyl transferase (GGT), total bilirubin (TBIL), direct bilirubin (DBIL), cholinesterase, serum albumin, prothrombin time, and the international normalized ratio (INR) for a comprehensive evaluation of liver injury.^[9,21]^

Previous studies have shown that severe liver injury often leads to alterations or interruptions in ART for HIV/AIDS patients,^[22]^ resulting in an increased disease burden,^[12]^ higher hospitalization rates,^[9]^ and poorer clinical outcomes.^[23]^ Early monitoring of the factors influencing severe liver injury and timely intervention are crucial for improving the clinical outcomes of HIV/AIDS patients with severe liver injury.^[3]^

Despite previous research^[16,23]^ primarily focusing on liver injury in patients undergoing ART, the factors influencing severe liver injury in ART-naïve HIV/AIDS outpatients remain unclear and may differ from those on long-term ART. Therefore, the objective of this study was to estimate the prevalence of liver injury in newly treated HIV/AIDS patients and to analyze the factors associated with severe liver injury in this population, in order to facilitate the screening of high-risk individuals and to develop optimized, individualized ART regimens.

## 2. Methods

### 2.1. Study design and patient inclusion

We conducted a retrospective analysis of HIV/AIDS patients who had established records in the outpatient department of the Infection Department at the Second Hospital of Nanjing from January 1, 2005, to May 31, 2019, but had not yet received ART. By integrating data from the national AIDS basic prevention and control information system database, the electronic medical record system of the Second Hospital of Nanjing, and paper medical records, we identified patients with liver injury prior to ART initiation.

The inclusion criteria were as follows: age ≥ 18 years old, with no gender restrictions; confirmed HIV/AIDS patients who met the health industry standard of the People’s Republic of China (WS293-2019) for the diagnosis of AIDS and HIV infection; attendance at the Infection Department clinic of the Second Hospital of Nanjing between January 1, 2005, and May 31, 2019; and evidence of liver injury before ART initiation, defined as ALT ≥ 1.25 × upper normal limit (ULN) and/or AST ≥ 1.25 × ULN and/or TBIL ≥ 1.1 × ULN, where the ULN was defined as ALT = 40U/L, AST = 40U/L, and TBIL = 19µmol/L. Patients missing any baseline ALT, AST, or TBIL results were excluded.

This study was approved by the Ethics Committee of the Second Hospital of Nanjing (2023-LS-013).

### 2.2. Definition and grouping

In this study, the baseline was defined as the date before ART initiation. According to the severity scale of adverse events in adults and children with AIDS, the liver injury grade of patients was determined using the highest grade of ALT, AST or TBIL value. Liver injury grades were assigned as follows: when ALT and/or AST levels reached 1.25 to 2.5, 2.5 to 5.0, 5.0 to 10.0, or ≥10.0 times ULN, and/or when TBIL levels reached 1.1 to 1.6, 1.6 to 2.6, 2.6 to 5.0, or ≥ 5.0 times ULN, they were classified as liver injury grade 1, 2, 3, and 4, respectively. Grades 1 and 2 were defined as mild liver injury (control group, Group1), and grades 3 and 4 were defined as severe liver injury (observation group, Group2).

Excessive alcohol consumption was defined as alcohol intake equivalent to ≥ 30 g/d and/or ≥210g/wk for men, and ≥ 20 g/d and/or ≥ 140 g/wk for women. Hepatitis B virus (HBV) infection was defined as hepatitis B surface antigen (HBsAg) positive and/or HBV deoxyribonucleic acid (HBV-DNA) positive. Hepatitis C virus (HCV) infection was defined as positive for HCV ribonucleic acid (HCV-RNA) with or without being positive for HCV antibodies. Concomitant disease was defined as the presence of diseases other than hepatitis B and C at baseline.

### 2.3. Clinical and laboratory data collection

Demographic data (age, sex, marital status, HIV transmission route, alcohol consumption), anthropometric indicators (height, weight, body mass index [BMI], and laboratory test results (CD4 + T cell count, HIV-1 viral load [VL]) HBsAg, HBV-DNA, hepatitis C antibody, HCV-RNA, triglycerides [TG], total cholesterol [TC], low-density lipoprotein cholesterol [LDL-C], high-density lipoprotein cholesterol [HDL-C], fasting blood glucose [FBG], alkaline phosphatase [ALP], gamma-glutamyl transferase [GGT], lactate dehydrogenase [LDH], ALT, AST, TBIL), comorbidities, and baseline clinical symptoms or signs were retrospectively collected from the National AIDS Basic Prevention and Control Information System database, the electronic medical record system of the Second Hospital of Nanjing, and paper medical records of patients.

AIDS-related disease was defined as the presence of at least one of the following conditions: thrush, persistent diarrhea for more than 1 month, skin lesions, oral hairy leukoplakia, persistent or intermittent fever for 1 month, recurrent or chronic upper respiratory tract infections, recurrent and severe bacterial infections (except pneumonia), recurrent and severe bacterial pneumonia, tuberculosis, extrapulmonary tuberculosis, disseminated nontuberculous mycobacterium infection, Mycobacterium avium complex infection, lymphocytic interstitial pneumonia, cryptococcal infections (including meningitis), toxoplasmosis, HIV encephalopathy, herpes zoster, chronic herpes simplex virus infection, cytomegalovirus infection, pneumocystis pneumonia, esophageal candidiasis, disseminated mycosis, brain lymphoma or B-cell non-Hodgkin lymphoma, Kaposi sarcoma, and other opportunistic infections.

### 2.4. Statistical analysis

Quantitative data are presented as medians (interquartile range), while categorical data are expressed as frequencies and percentages. Comparisons between patients with mild liver injury and those with severe liver injury were conducted using the Mann–Whitney U test for continuous variables and Chi-square test or Fisher exact test for categorical variables. Univariate and multivariate logistic regression models were employed to assess the risk factors associated with severe liver injury, with results reported as odds ratio (OR), and 95% confidence intervals (95%CI). Prior to logistic regression analysis, a collinearity diagnosis was performed on all variables to identify potential multicollinearity. A variance inflation factor >10 indicated severe multicollinearity. All tests were 2-tailed. Variables with a *P*-value < .05 in the univariate analysis were included in the multivariate logistic regression model. Statistical significance was set at *P* < .05 for all analyses. All statistical analyses were performed using SPSS version 26.0 (Chicago, IL).

## 3. Results

### 3.1. Overall demographic and clinical characteristics

**Figure 1.**
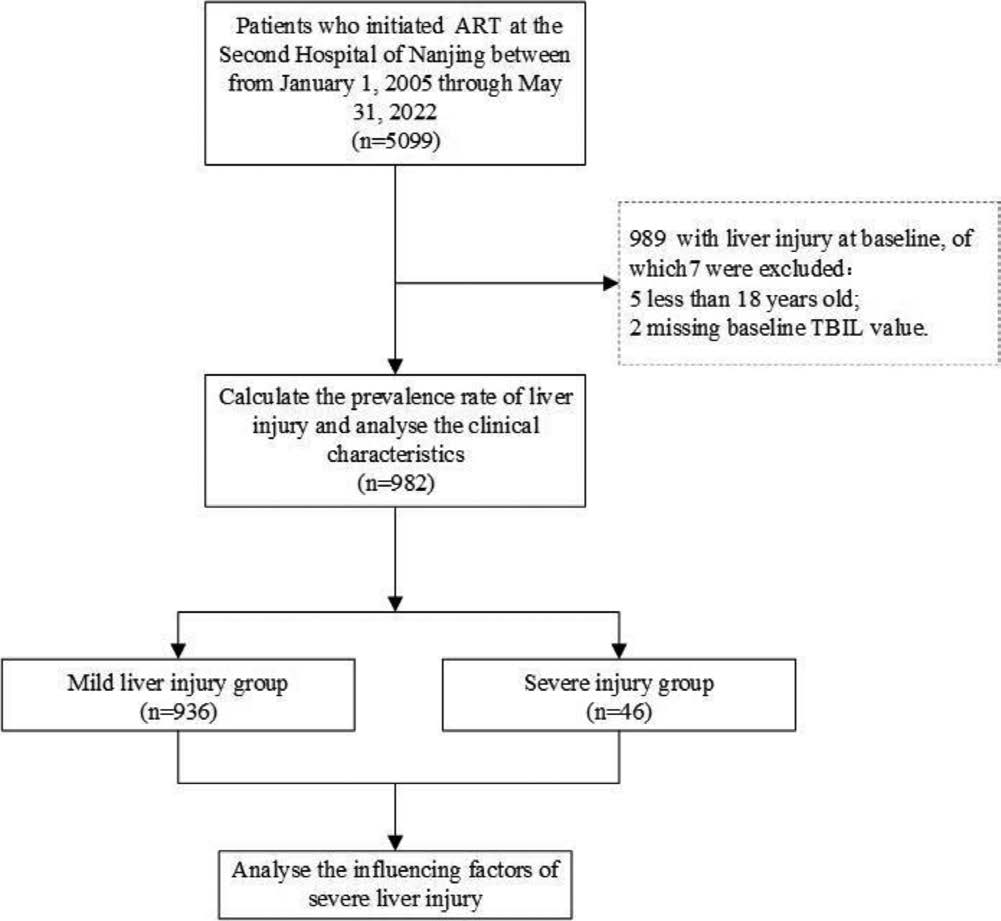
Study population flow diagram. This figure shows the screening process and inclusion criteria for the study population. Out of 5099 newly treated HIV/AIDS patients from January 1, 2005, to May 31, 2022, a total of 982 patients met the inclusion criteria for liver injury before ART initiation. Among them, 936 were classified into the mild liver injury group, while 46 were classified into the severe liver injury group. AIDS = acquired immunodeficiency syndrome, ART = antiretroviral therapy, HIV = human immunodeficiency virus.

A total of 5099 newly treated HIV/AIDS patients were screened from January 1, 2005, to May 31, 2022, in the Infection Department of the Second Hospital of Nanjing. Based on baseline levels of ALT, AST, and TBIL, 982 (19.3%, 982/5099) cases met the inclusion criteria and did not meet the exclusion criteria, and were thus included in the analysis (Fig. 1). Among these, 936 (95.3%) were classified into the mild liver injury group, while 46 (4.7%) were classified into the severe liver injury group (Fig. 1).

The median age of patients with liver injury was 32.0 years (IQR 26.0–32.0). The cohort was predominantly male, comprising 941 patients (95.8%), with a median BMI of 22.2 kg/m^2^ (IQR 20.0–24.6). Half of the patients (500, 50.9%) were unmarried, and 704 (71.7%) identified as men who have sex with men. The median CD4 + T cell count was 266.0cells/µL (IQR 76.0–400.3), and the median VL was 4.7 Log_10_ copies/mL (IQR 4.2–5.1). Excessive alcohol consumption was reported in 39 patients (4.0%). At baseline, 98 patients (10.0%) had HBV infection, 28 patients (2.9%) had HCV infection, and 285 patients (29.0%) had other comorbidities. Grade 1 liver injury was observed in 745 patients (75.9%), while 46 patients (4.7%) had grade 3 or higher liver injury (Table 1).

**Table 1:** Baseline demographic and clinical characteristics of ART-naïve HIV/AIDS patients with liver injury (N = 982).

Characteristics	General population	Mild liver injury group (936)	Severe liver injury group (46)	z/χ^2^	*P*
Age (IQR), yr	32.0 (26.0–32.0)	32.0 (26.0–42.0)	33.5 (27.8–50.0)	4.92	**.03**
＜50	837 (85.2)	803 (82.8)	34 (73.9)		
≥50	145 (14.8)	133 (14.2)	12 (26.1)		
Gender, No. (%)*				0.19	.66
Female	41 (4.2)	38 (4.1)	3 (6.5)		
Male	941 (95.8)	898 (95.9)	43 (93.5)		
BMI (IQR), kg/m^2^	22.2 (20.0–24.6)	22.2 (20.0–24.6)	22.3 (20.0–25.0)	3.58	.31
＜18.5, No. (%)	121 (12.3)	114 (12.2)	7 (15.2)		
18.5 to 24, No. (%)	544 (55.4)	519 (55.4)	25 (54.3)		
≥24, No. (%)	303 (30.9)	291 (31.1)	12 (26.1)		
NA, No. (%)	14 (1.4)	12 (1.3)	2 (4.3)		
Marital status, No. (%)				2.87	.24
Unmarried	500 (50.9)	480 (51.3)	20 (43.5)		
Married	402 (40.9)	378 (40.4)	24 (52.2)		
NA	80 (8.1)	78 (8.3)	2 (4.3)		
Transmission route, No. (%)				4.77	.09
Homosexual, No. (%)	704 (71.7)	676 (72.2)	28 (60.9)		
Heterosexual, No. (%)	215 (21.9)	199 (21.3)	16 (34.8)		
Others	63 (6.4)	61 (6.5)	2 (4.3)		
CD4+ T cell count (IQR), cell/μL	266.0 (76.0–400.3)	269.5 (77.3–402.0)	163.5 (46.3–311.0)	13.34	**<.001**
＜200, No. (%)	388 (39.5)	358 (38.2)	30 (65.2)		
≥200, No. (%)	594 (60.5)	578 (61.8)	16 (34.8)		
log_10_ VL (IQR), copies/mL	4.7 (4.2–5.1)	4.7 (4.2–5.1)	5.0 (4.7–5.4)	3.04	.22
＜100,000, No. (%)	470 (47.9)	452 (48.3)	18 (39.1)		
≥100,000, No. (%)	238 (24.2)	222 (23.7)	16 (34.8)		
NA	274 (27.9)	262 (28.0)	12 (26.1)		
Alcohol consumption, No. (%)				9.13	**.01**
Non-excessive drinking	642 (65.4)	621 (66.3)	21 (45.7)		
Excessive drinking	39 (4.0)	35 (3.7)	4 (8.7)		
NA	301 (30.7)	280 (29.9)	21 (45.7)		
Hepatitis B virus infection, No. (%)†				16.71	**<.001**
No	861 (87.7)	830 (88.7)	31 (67.4)		
Yes	98 (10.0)	84 (9.0)	14 (30.4)		
NA	23 (2.3)	22 (2.4)	1 (2.2)		
Hepatitis C virus infection, No. (%)†				0.13	1.00
No	932 (94.9)	888 (94.9)	44 (95.7)		
Yes	28 (2.9)	27 (2.9)	1 (2.2)		
NA	22 (2.2)	21 (2.2)	1 (2.2)		
Comorbidity, No. (%)				0.78	.38
No	697 (71.0)	667 (71.3)	30 (65.2)		
Yes	285 (29.0)	269 (28.7)	16 (34.8)		
AIDS-related diseases, No. (%)				2.38	.31
No	473 (48.2)	363 (48.7)	110 (46.4)		
Yes	429 (43.7)	317 (42.6)	112 (47.3)		
NA	80 (8.1)	65 (8.7)	15 (6.3)		
TG (IQR), mmol/L	1.3 (1.0–1.9)	1.3 (0.9–1.9)	1.4 (1.2–1.7)	0.73	.47
TC (IQR), mmol/L	4.7 (4.1–5.4)	4.1 (3.4–4.7)	3.8 (3.0–4.6)	1.37	.17
LDL-C (IQR), mmol/L	2.4 (1.9–3.0)	2.4 (1.9–3.0)	2.1 (1.6–3.1)	1.26	.21
HDL-C (IQR), mmol/L	1.2 (1.0–1.4)	1.0 (0.8–1.2)	0.8 (0.4–1.0)	3.99	**<.001**
FBG (IQR), mmol/L	5.3 (4.9–5.8)	5.3 (4.9–5.8)	5.4 (4.8–6.4)	0.64	.53
ALP (IQR), IU/L	90.2 (69.9–123.1)	69.0 (57.0–88.1)	96.2 (63.5–140.1)	4.00	**<.001**
GGT (IQR), U/L	74.2 (37.3–130.8)	36.2 (20.8–66.4)	104.2 (53.4–181.9)	6.31	**<.001**
LDH (IQR), IU/L	274.8 (223.0–348.2)	221.0 (189.0–268.8)	317.0 (256.5–435.8)	6.27	**<.001**
Grade of liver injury, No. (%)†				358.62	**<.001**
Grade 1	745 (75.9)	745 (79.6)	0 (0.0)		
Grade 2	191 (19.5)	191 (20.4)	0 (0.0)		
Grade 3	34 (3.5)	0 (0.0)	34 (73.9)		
Grade 4	12 (1.2)	0 (0.0)	12 (26.1)		

The bold values indicate statistically significant results (*P* < .05).

AIDS = acquired immunodeficiency syndrome, ALP = alkaline phosphatase, ART = antiretroviral therapy, BMI = body mass index, FBG = fasting blood glucose, GGT = gamma-glutamyl transpeptidase, HDL-C = high-density lipoprotein cholesterol, HIV = human immunodeficiency virus, IQR = interquartile range, LDH = lactic dehydrogenase, LDL-C = low-density lipoprotein cholesterol, NA = not available, TC = total cholesterol, TG = triglyceride, VL = viral load.

* Corrected chi-square.

† Fisher exact probability method.

### 3.2. Comparison between groups of patients with mild and severe liver injury

Gender, marital status, HIV infection route and alcohol consumption were similar between the mild and severe liver injury groups (all *P* > .05). Compared to the mild liver injury group, patients in the severe liver injury group were older at baseline [33.5 (27.8–50.0) vs 32.0 (26.0–42.0), years, *P* = .03]. The CD4 + T cell count was significantly lower [207.0 (38.5–364.5) vs 272.0 (89.9–402.5), cells/µL, *P* < .001], and the proportion of patients with excessive alcohol consumption was higher [8.7% (4/46) vs 3.7% (84/936). *P* = .01]. Additionally, a higher percentage of patients in the severe liver injury group were infected with HBV [30.4% (14/46) vs 9.0% (84/936), *P* < .001]. HDL-C levels were lower in the severe liver injury group [0.8 (0.41.0) vs 1.0 (0.8–1.2), IU/L] while ALP, GGT, and LDH levels were significantly higher [96.2 (63.5–140.1) vs 69.0 (57.0–88.1), IU/L], [104.2 (53.4–181.9) vs 36.2 (20.8–66.4), U/L], [317.0 (256.5–435.8) vs 221.0 (189.0–268.8), IU/L], with all differences statistically significant (*Z* = 3.99, 4.00, 6.31, 6.27, all *P* < .001). There were no statistically significant differences in baseline VL, HCV infection status, comorbidities, TG, TC, LDL-C and FBG between the 2 groups (all *P* > .05), as shown in Table 1. At baseline, 492 patients (43.7%) exhibited clinical symptoms or signs, including 317 patients (42.6%) with mild liver injury and 112 patients (47.3%) with severe liver injury, with no significant difference between the 2 groups (χ^2^ = 2.38, *P* = .31).

### 3.3. Abnormal level distribution of liver function indicators

**Figure 2.**
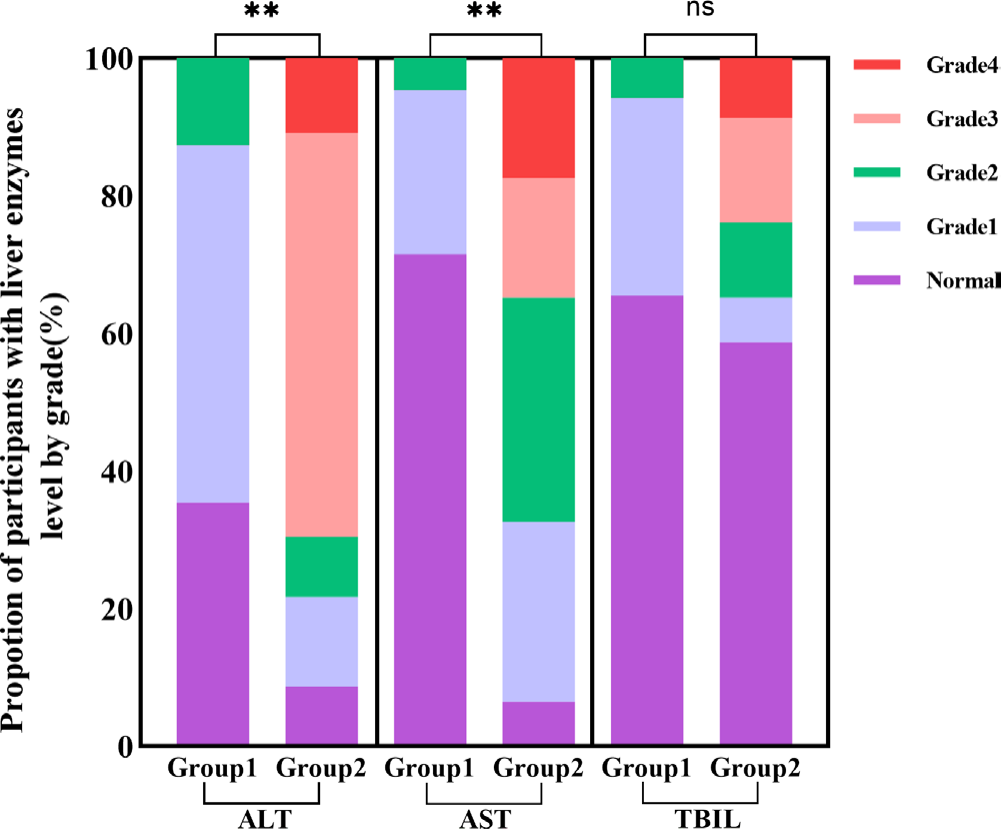
Distribution of abnormal liver function indicators. The figure illustrates the proportion of patients with abnormal levels of ALT, AST, and TBIL in the mild and severe liver injury groups. At baseline, the proportion of patients with abnormal ALT and AST levels was significantly higher in the severe liver injury group compared to the mild liver injury group. However, the proportion of patients with abnormal TBIL levels was similar between the 2 groups. ALT = alanine aminotransferase, AST = aspartate aminotransferase, TBIL = total bilirubin.

At baseline, the proportion of patients with abnormal ALT levels was significantly higher in the severe liver injury group compared to the mild liver injury group [Group1 vs Group2, 605 (64.6%) vs 42 (91.3%), χ^2^ = 13.87, *P* < .001]. Similarly, the proportion of patients with abnormal AST levels was significantly higher in the severe liver injury group [Group1 vs Group2, 267 (28.5%) vs 43 (93.5%), χ^2^ = 85.63, *P* < .001]. However, the proportion of patients with abnormal TBIL levels was similar between the 2 groups [Group1 vs Group2, 323 (34.5%) vs 19 (41.3%), χ^2^ = 0.89, *P* = .35] (Fig. 2).

### 3.4. Logistic regression analysis of severe liver injury factors

Univariate analysis of HIV/AIDS patients with moderate and severe liver damage at baseline indicated several significant factors associated with the occurrence of severe liver injury. These factors included baseline age ≥ 50 years (OR = 2.13, 95% CI = 1.08–4.22, *P* = .03), CD4 + T cell count < 200cells/µL (OR = 3.03, 95% CI = 1.63–5.63, *P* < .001), excessive alcohol consumption (OR = 3.38, 95% CI = 1.10–10.38, *P* = .03), HBV infection (OR = 4.46, 95% CI = 2.28–8.72, *P* < .001), decreased HDL-C (OR = 0.06, 95% CI = 0.02–0.12, *P* < .001), increased ALP (OR = 1.05, 95% CI = 1.02–1.08, *P* = .001), increased GGT (OR = 1.06, 95% CI = 1.03–1.08, *P* < .001), and elevated LDH (OR = 1.03, 95% CI = 1.02–1.05, *P* < .001) (Table 2).

**Table 2:** Logistic regression analysis of liver injury in HIV/AIDS patients with baseline liver injury.

	Univariate analysis		Multivariate analysis	
	OR (95% CI)	*P*	OR (95% CI)	*P*
Age (IQR), years				
＜50	1.00			
≥50	2.13 (1.08–4.22)	**.03**	1.59 (0.67–3.79)	.30
CD4 + T cell count, cell/mL				
≥200	1.00		1.00	
＜200	3.03 (1.63–5.63)	**＜.001**	1.21 (0.55–2.64)	.64
Alcohol consumption				
Non-excessive drinking	1.00			
Excessive drinking	3.38 (1.10–10.38)	**.03**	2.49 (0.66–9.35)	.18
Hepatitis B virus infection				
No	1.00			
Yes	4.46 (2.28–8.72)	**＜.001**	4.02 (1.82–8.88)	**.001**
HDL-C, mmol/L	0.06 (0.02–0.20)	**＜.001**	0.28 (0.08–0.99)	**.048**
ALP*, per 10U/L increase	1.05 (1.02–1.08)	**.001**		
GGT, per 10U/L increase	1.06 (1.03–1.08)	**＜.001**	1.04 (1.01–1.06)	**.004**
LDH, per 10IU/L increase	1.03 (1.02–1.05)	**＜.001**	1.03 (1.01–1.04)	**.002**

The bold values indicate statistically significant results (*P* < .05).

AIDS = acquired immunodeficiency syndrome, ALP = alkaline phosphatase, CI = confidence interval, GGT = gamma-glutamyl transpeptidase, HDL-C = high-density lipoprotein cholesterol, IQR = interquartile range, LDH = lactic dehydrogenase, OR = odds ratio.

* Multiple Logistic regression was not included in ALP due to much missing data (268/982).

Factors with *P* < .05 in the univariate analysis were included in the multivariate logistic regression model. ALP was excluded from the multivariate analysis due to excessive missing values (27.3%, 268/982) (Table 2). The multivariate logistic regression model analysis indicated that HDL-C (OR = 0.28, 95% CI = 0.08–0.99, *P* = .048) was a protective factor for severe liver injury. Conversely, HBV infection (OR = 4.02, 95% CI = 1.82–8.88, *P* = .001), increased GGT (OR = 1.04, 95% CI = 1.01–1.06, *P* = .004) and elevated LDH (OR = 1.03, 95% CI = 1.01–1.04, *P* = .002) were identified as independent risk factors for severe liver injury in newly treated HIV/AIDS patients. Age ≥ 50 years (OR = 1.59, 95% CI = 0.67–3.79, *P* = .30), CD4 + T cell count < 200cells/µL (OR = 1.21, 95% CI = 0.55–2.64, *P* = .64), and excessive alcohol consumption (OR = 2.49, 95% CI = 0.66–9.35, *P* = .18) were not significantly associated with severe liver injury (Table 2).

## 4. Discussion

Our study provides novel insights into the prevalence and influencing factors of liver injury among ART-naïve HIV/AIDS patients. We found that 19.3% of these patients experienced liver injury, with HDL-C identified as a protective factor and HBV infection, elevated GGT, and LDH as significant risk factors for severe liver injury. These findings contribute new knowledge by identifying specific biomarkers and co-infections influencing liver injury severity in a population that has not yet initiated ART.

Liver injury is a common complications in HIV-infected patients, significantly affecting the selection and efficacy of ART regimen. Severe liver injury may also lead to poor prognosis.^[9]^ According to Smith C.J. et al., the mortality rate of liver-related diseases in HIV patients is as high as 10.2%, making it the second leading cause of non-AIDS-related deaths.^[24]^ However, there are few studies on liver injury in ART-naïve individuals, and the factors contributing to severe liver injury remain largely unknown.

In this study, the prevalence rate of liver injury in HIV/AIDS patients before ART was 19.3%, with the majority of cases being mild (75.9%), consistent with previous studies,^[10]^ indicating that HIV infection itself could cause liver injury. However, the overall prevalence of liver injury in this study (19.3%) was significantly higher than that reported by Nagu,T.J. et al.(13%).^[10]^ This discrepancy may be attributed to the gender composition of the included patients (60.5% male and 39.5% female in this study).^[10]^ In contrast, the prevalence of liver injury at baseline was higher in studies from Cameroon (42.1%)^[10]^ and China (27.23%),^[25]^ suggesting that long-term exposure to antiretrovirals (ARVs) may increase the risk of liver injury.

In our study, the prevalence of hepatitis B infection at baseline was 10.0%, consistent with the findings of Jing Xie et al.^[26]^ in a Chinese study. After multivariate analysis, baseline HBV positivity remained an independent risk factor for severe liver injury in HIV/AIDS patients prior to ART. This may be due to HIV-induced immunodeficiency enhancing HBV-related hepatotoxicity mediated by immune responses.^[27]^ Depletion of CD4 + T cells, a hallmark of HIV infection,^[28]^ inhibits antigen presentation by liver macrophages (Kupffer cells) and cytokine secretion by lymphocytes, leading to host immunosuppression.^[29]^ The suppression of the host immune response by HIV infection significantly enhances HBV replication,^[30]^ which may in turn leads to severe liver damage.

This study identified HDL-C as a protective factor against severe liver injury in HIV/AIDS patients prior to ART initiation through multivariate analysis. The baseline median HDL-C in the group with severe liver injury was below the lower limit of the normal reference range, suggesting a potential correlation between HDL-C decline and severe liver injury. Numerous studies have observed similar findings in HIV patients; for instance, Spaziante et al.^[31]^ reported reduced levels of TC and HDL-C in HIV-infected patients before ART. The Multicenter AIDS Cohort Study (MACS) noted decreases in TC, HDL-C, and LDL-C levels following HIV seroconversion,^[32]^ while TC and LDL-C levels increased post-ART initiation, HDL-C levels continued to decline. The connection between HDL-C reduction and severe liver injury, however, remains inadequately explained. One possibility is that HIV infection itself leads to excessive visceral adipose tissue accumulation in patients, with HIV-associated fat redistribution impacting lipid metabolism in hepatocytes.^[33]^ Another consideration is that patients with severe liver injury may exhibit abnormal lifestyle behaviors, contributing to an abnormal lipid profile. We hypothesize that the observed association between reduced HDL-C levels and severe liver injury may stem from metabolic disturbances induced by HIV infection and lifestyle abnormalities in patients.

This study identified a strong correlation between GGT and LDH with severe liver injury through multifactorial analysis. We hypothesized that this phenomenon could be explained by oxidative stress and the effects of HIV infection itself. In the liver, the primary immune cells are stellate cells (HSCs) and Kupffer cells. Kupffer cells activate local inflammatory responses and facilitate hepatocyte repair, while HSCs are major contributors to liver fibrosis and extracellular matrix protein deposition.^[34]^ Oxidative stress involves the activation of Kupffer cells by reactive oxygen species (ROS). Nuclear factor kappa B (NF-kB) and activating protein 1 promote the activation of HSCs, leading to increased production of pro-inflammatory and pro-fibrotic cytokines.^[35]^ If left unchecked, this process can result in liver damage, fibrosis and cirrhosis.^[36]^ Various factors, including alcohol, viral hepatitis, nonalcoholic fatty liver disease (NAFLD), HIV, and certain drugs, have been shown to induce liver damage through this mechanism.^[3]^ Additionally, HIV and its envelope protein gp120 interact with C-C chemokine receptor type 5 (CCR5) and C-X-C chemokine receptor type 4 (CXCR4) on the surface of hepatocytes, HSCs and other immune cells,^[37]^ thereby accelerating hepatocyte apoptosis. The increase in ROS leads to the formation of oxidized glutathione (GSSH) from reduced glutathione (GSH) in liver cells. Membrane-bound GGT, a key enzyme in the gamma-glutamyl cycle, degrades bound glutathione through transpeptidation, recycling the metabolites back into the cycle.^[38]^ In the event of severe liver injury, widespread hepatocyte apoptosis releases GGT into the bloodstream, resulting in elevated serum GGT levels. Furthermore, a large 6-year cohort study^[39]^ in Germany found that elevated GGT levels were more sensitive predictor of liver decompensation, hepatocellular carcinoma (HCC), and death than ALT, regardless of the presence of cirrhosis.

Mitochondrial products are the primary source of energy for liver cells. Consequently, any process impairing mitochondrial function may lead to liver damage.^[40]^ Mitochondrial dysfunction results in an increase in ROS, which, while involved in cell signaling, can also induce toxicity and DNA damage. This leads to the decoupling of mitochondria-mediated respiration from adenosine triphosphate (ATP) synthesis, subsequently increasing mitochondrial oxygen consumption and fatty acid oxidation.^[41]^ Proteins encoded by HIV-1 virus, including Vpr, Nef and Vpu, can mediate HIV-induced apoptosis through mitochondrial activity.^[42]^ We hypothesized that the elevated serum LDH levels observed in HIV patients may be attributed to the insufficient energy supply to liver cells due to mitochondrial damage, prompting a shift from aerobic metabolism to anaerobic metabolism (glycolysis) and resulting in increased lactic acid production. LDH catalyzes the conversion of pyruvate to lactic acid, and its levels rise when anaerobic metabolism is enhanced.^[43]^ Further investigation into the relationship between lactate and LDH values in newly treated HIV patients with liver injury is required to verify this hypothesis.

This study did not find an association between CD4 + T cell count of <200cells/µL and severe liver injury. The conclusions reported in different studies are also inconsistent.^[44,45]^ This inconsistency may be related to the fact that enhanced immunity can adversely affect diseases involving immune-mediated mechanisms, such as autoimmune diseases or chronic viral hepatitis, which may lead to elevated liver enzymes. Conversely, enhanced immunity can reduce opportunistic infections during HIV infection, resulting in decreased liver enzyme levels.^[46]^

Unlike previous studies,^[9]^ this study did not find an association between excessive alcohol consumption and severe liver injury. This may be due to the small number of self-reported cases of excessive alcohol consumption in our study population (4.0%). Additionally, the number of individuals infected with hepatitis C in our population is small, and the low infection rate (2.9%) may not be sufficient to detect an association between hepatitis C infection and severe liver injury. Furthermore, data on excessive alcohol consumption and HCV infection were missing to varying degrees in our study, which may also contribute to the lack of observed association between these factors and severe liver injury.

Our study possesses several notable strengths. First, it utilizes a large sample size, which enhances the statistical power and reliability of our findings. Second, while previous research has predominantly focused on liver injury in patients undergoing ART, often attributing severe liver injury to ART toxicity, our study demonstrates that significant liver injury can occur even before ART initiation. Our finding that HDL-C serves as a protective factor is consistent with limited prior research but offers a more robust statistical validation through multivariate analysis. Additionally, the identification of HBV infection and elevated GGT and LDH as risk factors aligns with earlier studies but extends these findings to the ART-naïve population. These results have important implications for clinical management and public health strategies. Early identification and monitoring of these risk factors can help clinicians stratify risk and tailor interventions to prevent severe liver injury in HIV/AIDS patients. This proactive approach could improve patient outcomes by reducing the burden of liver-related morbidity and mortality in this vulnerable population. Future research should focus on longitudinal studies to confirm these findings and explore the mechanisms underlying the protective role of HDL-C and the harmful effects of HBV infection and elevated GGT and LDH. Additionally, investigating the impact of early intervention strategies on liver injury outcomes in ART-naïve patients could provide valuable insights for clinical practice.

Our study also has several limitations. Being cross-sectional, it could not account for all potential confounders; therefore, these observations need to be confirmed in a prospective cohort. Additionally, some indicators in the study, such as HIV-1 viral load, alcohol consumption, and ALP deletion value, were high, which may affect the results. However, these factors were not included in the model when multivariate logistic regression was performed. Moreover, this study defined the threshold for liver injury as 1.25 × ULN rather than exceeding ULN, which may be an underestimate the prevalence of mild liver injury.

## 5. Conclusion

In summary, the baseline prevalence of liver injury among newly treated HIV/AIDS patients at the Second Hospital of Nanjing was 19.3% (982/5099), with the majority of cases being mild in severity. HDL-C was a protective factor against severe liver injury. Conversely, HBV infection, elevated GGT levels and increased LDH levels were found to be independent risk factors for severe liver injury.

## Acknowledgments

We extend our gratitude to all patients who participated in our study, as well as the investigators and site staff for their dedicated efforts. We also thank the AIDS Healthcare Foundation for their support of our work.

## Author contributions

**Conceptualization:** Nawei Yu, Hongxia Wei.

**Data curation:** Nawei Yu.

**Formal analysis:** Nawei Yu.

**Funding acquisition:** Hongxia Wei.

**Investigation:** Nawei Yu, Xiaoyun Di, Zihao Xia, Jingli Peng, Mengqing Li, Hongjing Guan.

**Project administration:** Chen Chen, Rentian Cai, Hongxia Wei.

**Resources:** Nawei Yu.

**Supervision:** Hongxia Wei.

**Validation:** Nawei Yu, Mingli Zhong.

**Visualization:** Nawei Yu.

**Writing – original draft:** Nawei Yu.

**Writing – review & editing:** Nawei Yu, Mingli Zhong, Hongxia Wei.
